# Health Literacy and Regional Heterogeneities in China: A Population-Based Study

**DOI:** 10.3389/fpubh.2021.603325

**Published:** 2021-05-11

**Authors:** Zhenhua Li, Yongquan Tian, Zhicheng Gong, Long Qian

**Affiliations:** ^1^Department of Social Medicine and Health Management, School of Public Health, Central South University, Changsha, China; ^2^Xiangya Hospital, Central South University, Changsha, China

**Keywords:** health literacy, regional heterogeneities, health literacy scale, distribution characteristics, influencing factors

## Abstract

**Background:** Health literacy is essential to population health, yet few studies have described the geographic variation in health literacy in China. This study aimed to investigate the level of health literacy, its regional heterogeneities, as well as influencing factors of health literacy in 25 provinces or municipalities in China.

**Methods:** The study was conducted among residents aged 15–69 years from 25 provinces or municipalities in China in 2017. Health literacy was measured using the Chinese Health Literacy Scale. MapInfo software was used to map the geographic distribution. Multiple logistic regression was used to adjust for the factors associated with the health literacy level in the overall and regional samples.

**Results:** A total of 3,482 participants were included in the study, comprising 1,792 (51.5%) males and 1,690 (48.5%) females. Notable geographic variation was observed in health literacy levels. The proportion of respondents with adequate health literacy was 22.3% overall, 33.0% in the eastern region, 23.1% in the central region, and 17.6% in the western region. The proportion of adequate health literacy in the different provinces and municipalities ranged from 10.5% (Xinjiang) to 47.0% (Beijing). Being a female [odds ratio (OR) = 1.353; 95% confidence interval (CI): 1.146–1.597], having a high education level [OR ranging from 2.794 (CI: 1.469–5.314) to 9.458 (CI: 5.251–17.036)], having a high economic status [OR ranging from 1.537 (CI: 1.248–1.891) to 1.850 (CI: 1.498–2.284)], having a good self-rated health status [OR ranging from 2.793 (CI: 1.534–5.083) to 3.003 (CI: 1.672–5.395)], and having frequent community health education (OR = 1.588; 95% CI: 1.066–2.365) were independently associated with adequate health literacy.

**Conclusions:** The health literacy level in the 25 provinces or municipalities of China is relatively low compared to the developed countries, and there are heterogeneities among different regions, between urban and rural areas, and among different social groups. Tailored health education and promotion strategies are needed for different subgroups of residents.

## Introduction

Health literacy refers to the ability of individuals to acquire and understand basic health information and services and to use them to make informed decisions to maintain and promote their health (1). The definition of health literacy that has been proposed by the WHO was designed to include the promotion and improvement of individual and community health (2). In different countries, the measurement tools and research perspectives of health literacy are different, and the standards are not uniform (3).

Many studies with various methodologies have shown that deficiencies in health literacy are related to poor life expectancy and quality of life, poor healthcare utilization and health outcomes (relatively high mortality rates and poor overall health status), and health disparities (4–7). The economic implications of low health literacy are substantial, with some estimates accounting for up to 5% of health care costs annually (8). People with limited health literacy may not properly understand health information from health practitioners or the media, and cannot effectively utilize healthcare (9); these deficiencies may be associated with reduced life expectancy and increased health care costs (9). Improvements in health literacy are an effective and easy way to improve health (10). Governments and national agencies in the USA, China, Australia, and some European countries have developed national strategies and targets to improve health literacy in their populations (11).

Health literacy research began late in China. In 2008, based on research results and experiences pertaining to health literacy at home and abroad, the former Ministry of Health of China organized medical and health experts to define the 66 basic components of Chinese health literacy and compiled the Chinese Health Literacy Monitoring Questionnaire. In the same year, the first survey of health literacy was conducted nationwide. The survey results showed that the overall level of health literacy among Chinese people was 6.48% (12).

China covers a vast geographical area, divided into three geographical regions: the eastern region, the central region, and the western region, and the conditions in different regions vary widely (13). The level and status of economic and social development differ on a regional basis (13). Additionally, health disparities persist among China's three geographic regions: eastern, central and western (14). Health outcomes are generally poorer in the western region than in the central or eastern region (13). Additionally, the level of health literacy is affected by social factors, such as the economy and culture (15).

Few studies have described the geographic variation in health literacy in China. This study aimed to investigate the levels of health literacy in Chinese residents from 25 provinces or municipalities and the heterogeneity of health literacy among regions. This information will provide scientific evidence to facilitate tailored health promotion strategies in different economic and cultural contexts.

## Materials and Methods

### Study Design

This was a cross-sectional study of health literacy and its geographic heterogeneity in 25 provinces or municipalities of China, independent of the national monitoring survey. Health literacy was measured using the Chinese Health Literacy Scale. The study subjects were permanent residents aged 15–69 years who had continuously lived in the survey areas for more than 6 months. We excluded those aged below 15 because this age group usually haven't completed basic compulsory education yet. Residents with cognitive impairment or hearing loss were excluded from the study.

The research protocol was reviewed and approved by the Medical Ethics Committee of Central South University. All participants aged 16 and older who agreed to participate in the study signed an informed consent form at the beginning of the survey. Written informed consent was obtained from a parent or guardian for participants under 16 years old.

### Sampling Methods

We selected 25 provinces out of all 31 provincial administrative regions in mainland China. The other 6 provinces were not selected due to difficulty of getting support from the local governments and limited funding. The selected 25 provinces are diverse in geography, economic level, population etc. A multistage, stratified, probability proportional to size sampling was used. Based on the hierarchical administrative system and 2010 Chinese Census data (16), sampling was undertaken across the following five stages: (a) 2–3 counties were randomly selected in each province according to regional and population factors, (b) one street (township) was randomly selected within each county, (c) one community was randomly selected within each street (township), (d) 40–50 households were randomly selected from each community according to the community's resident roster, and (e) one eligible respondent was randomly selected from each selected household. The sample size (*N* = 2,419) was calculated to ensure a proportion estimation of adequate health literacy with α = 0.05 based on a conservative assumption of a 15% proportion.

### Study Measures

#### Demographic Characteristics

The socio-demographic characteristics collected in this study included gender (male or, female), age (15–29, 30–49, or 50–69 years), place of residence (eastern, central or western region), community type (urban or rural community), marital status (single or married), education level (elementary school and below, junior high school, senior high school, or college and above), and economic status (poor, medium, or good). The surveyed residences were divided into the eastern, central, and western regions according to the region classification in the China Health Statistics Yearbook. Economic status was divided into the poor, medium, and good categories, with the cutoff points being 75 and 125% of the median annual household income per capita.

#### Health Literacy

The Chinese Health Literacy Scale, prepared by the Chinese Center for Health Education, was used to measure health literacy. This scale assesses health Knowledge, attitudes, behaviors and skills that are necessary to address real-world health problems and consists of 6 dimensions (17). The overall Cronbach's alpha of the scale was 0.95, and the Spearman-Brown coefficient was 0.94 (18). Confirmatory factor analysis showed that the scale measured a unidimensional construct with three highly correlated factors (18): (a) basic knowledge and attitudes (BKA), (b) healthy lifestyles and behaviors (HLB), and (c) health-related skills (HRS). The scale covers six domains: scientific views of health (SVH), prevention and treatment of infectious diseases (PTID), prevention and treatment of chronic diseases (PTCD), safety and first aid (SFA), basic medical care (BMC), and health information (HI).

There are three types of questions on the scale: true or false (with 1 point given for each correct response), single answer (a multiple-choice question with only one correct answer, where 1 point is given for each correct response), and multiple answer (a multiple-choice question with more than one correct answer, where two points are for each correct response). For the multiple-answer questions, a correct response was defined as one that contained all of the correct answers and none of the incorrect ones.

The maximum total score of the scale is 66 points, with the maximum total scores of the three dimensions being 28 (BKA), 22 (HLB), and 16 (HRS) points. The maximum total scores for SVH, PTID, PTCD, SFA, BMC, and HI are 11, 7, 12, 14, 14, and 8 points, respectively.

A total score of 53 (80% of 66) points or above was considered to indicate adequate health literacy. A score of 0–52 was considered to indicate limited health literacy. The health literacy level was defined as the proportion of participants who had adequate health literacy out of the total number of participants. The judgment criterion for adequate health literacy in each dimension or domain was ≥80% of the total score for the dimension or domain (18, 19).

#### Health Status

The self-evaluated health status was used as the evaluation index and was divided into good, fair, and poor levels. The original question was, “What do you think of your health status in the past year?”

#### Community Health Education

We used the number of health lectures given by the primary care practitioners as a proxy measure of community health education, determined by a question, “How many health lectures did you attend in your community during the past three years?” The self-reported frequency of participation in community health education was divided into three categories (0 times, 1–9 times, and ≥10 times).

#### Survey Method

In the pre-investigation phase, a certain number of respondents were randomly selected from the sample locations for pre-surveys, focusing on whether the questionnaire items were unambiguous and clearly understood. The results showed that the respondents could understand the contents of the questionnaires. In the formal investigation phase, face-to-face interviews were conducted at each participants' home or other public places at the participants' convenience. Putonghua, which is China's uniform language was used in the interviews. For participants who did not understand Putonghua, one family member who could speak Putonghua was invited as interpreter for the interview. Information was collected using paper-based questionnaires by field investigators based on the interviews. In the re-testing phase, which was 2 weeks after the formal investigation, 155 respondents were randomly selected from the overall sample using a computer-based simple random sampling technique, and the investigators re-tested those subjects by phone. All phases of the investigation were conducted by trained investigators. Prior to the investigation, all investigators were given uniform training for this survey. The investigation was conducted from January to April 2017.

### Statistical Analyses

Statistical analysis was conducted with SPSS version 19.0 (IBM Corp., Armonk, NY, USA) and MapInfo Professional version 7.0 (Pitney Bowes MapInfo Corp., Stamford, USA). An integrity check was performed before submitting the questionnaire, and questionnaires with missing values were not included in the analysis. Prior to the analysis, data were screened for outliers and out-of-range values. No outliers or out-of-range values were found. The general conditions and health literacy of the sample were statistically described as the mean ± standard deviation, composition ratio, median, and frequency distribution table. In order to evaluate the factors of health literacy, the health literacy scores were dichotomized into two categories: adequate and limited. The chi-squared (χ^2^) test was used to compare the health literacy levels among different characteristic groups. The geographic variations of health literacy levels were described using MapInfo software, and the National Platform for Common Geospatial Information Services of China provided the map. A series of multiple logistic regressions was used to adjust for the relevant factors associated with the health literacy level in the total and regional samples. The logistic regression analyses were performed with gender, age group, marital status, community type, education level, economic status, self-rated health status, and frequency of participation in community health education as the independent variables; adequate health literacy served as the dependent variable in the overall and regional samples. An adequate health literacy equation was established using a multiple logistic regression model with stepwise forward selection. In all hypothesis tests, two-sided *P*-values of <0.05 were taken to indicate statistical significance.

## Results

### Basic Characteristics

Among the 3,600 surveyed people, 3,482 valid questionnaires without apparent logical errors or missing items were obtained, yielding an effective response rate of 96.7% (3,482/3,600) for the questionnaire. The test-retest reliability of the scale score was 0.953. The respondents included 566 (16.3%) individuals in the eastern region, 1,397 (40.1%) in the central region, and 1,519 (43.6%) in the western region (Table 1). The male: female ratio was 1.06:1, and the average age was 34.27 ± 13.72 years. The education level of the respondents was mainly college and above, accounting for 51.3% of the sample. The ethnic group was mainly Han, accounting for 81.5% of the sample. With respect to marital status, the majority of participants (57.7%) were married. The median annual income per capita was 20,000 CNY. A majority (60.6%) of the respondents had not participated in community health education within the past 3 years. No statistically significant difference was found in the gender composition (χ^2^ = 4.962, *P* = 0.084) or age composition (χ^2^ = 7.201, *P* = 0.126) of the respondents among the eastern, central, and western regions.

**Table 1:** Association between health literacy level and basic characteristics.

**Characteristics**	**Health literacy**		**Percentage (%)**	**χ^2^**	***P*-value**
	**Adequate HL (%)**	**Limited HL (%)**			
Gender				13.060	<0.001
Male	356 (45.8)	1,436 (53.1)	1,792 (51.5)		
Female	422 (54.2)	1,268 (46.9)	1,690 (48.5)		
Age group (years)				28.972	<0.001
15–29	392 (50.4)	1,192 (44.1)	1,584 (45.5)		
30–49	325 (41.8)	1,104 (40.8)	1,429 (41.0)		
50–69	61 (7.8)	408 (15.1)	469 (13.5)		
Region				57.142	<0.001
Eastern region	187 (24.0)	379 (14.0)	566 (16.3)		
Central region	323 (41.5)	1,074 (39.7)	1,397 (40.1)		
Western region	268 (34.4)	1,251 (46.3)	1,519 (43.6)		
Community type				8.700	0.003
Urban	488 (62.7)	1,536 (56.8)	2,024 (58.1)		
Rural	290 (37.3)	1,168 (43.2)	1,458 (41.9)		
Education level				174.930	<0.001
Elementary school and below	12 (1.5)	314 (11.6)	326 (9.4)		
Junior high school	55 (7.1)	487 (18.0)	542 (15.6)		
Senior high school	173 (22.2)	654 (24.2)	827 (23.8)		
College and above	538 (69.2)	1,249 (46.2)	1,787 (51.3)		
Marital status				3.621	0.058
Single	352 (45.2)	1,120 (41.4)	1,472 (42.3)		
Married	426 (54.8)	1,584 (58.6)	2,010 (57.7)		
Self-rated health status				23.071	<0.001
Good	559 (71.9)	1,795 (66.4)	2,354 (67.6)		
Fair	206 (26.5)	757 (28.0)	963 (27.7)		
Poor	13 (1.7)	152 (5.6)	165 (4.7)		
Economic status				65.537	<0.001
Good	294 (37.8)	704 (26.0)	998 (28.7)		
Medium	285 (36.6)	913 (33.8)	1,198 (34.4)		
Poor	199 (25.6)	1,087 (40.2)	1,286 (36.9)		
Community health education (frequency)				7.429	0.024
0	442 (56.8)	1,667 (61.6)	2,109 (60.6)		
1–9	296 (38.0)	937 (34.7)	1,233 (35.4)		
≥10	40 (5.1)	100 (3.7)	140 (4.0)		

*Adequate HL, adequate health literacy; limited HL, limited health literacy*.

### Distribution of Health Literacy

The univariate analysis showed significant differences in health literacy by gender, age, region, community type, education level, self-rated health status, economic status, and frequency of participation in community health education (Table 1). We found that school-age group (15–24) had significantly higher health literacy than above-school-age groups, indicating that school education can effectively promote health literacy.

The proportion of respondents with adequate health literacy was 22.3% (778/3,482) overall, 33.0% (187/566) in the eastern region, 23.1% (323/1,397) in the central region, and 17.6% (268/1,519) in the western region (Table 2). The proportions of BKA, HLB, and HRS were 42.2, 17.7, and 28.0%, respectively. From high to low, the proportions of health literacy in different dimensions were 62.7% for SFA literacy, 59.7% for SVH, 32.4% for HI, 23.5% for PTID, 23.4% for PTCD, and 22.9% for BMC. Except for PTID, statistically significant differences were found in all dimensions and domains of health literacy among individuals from different regions (Table 2).

**Table 2:** Percentage of participants with adequate health literacy in different regions by dimensions and domains.

**Dimensions/ domains**	**Eastern region**	**Central region**	**Western region**	**Total (*n* = 3,482) (%)**
	**(*n* = 566) (%)**	**(*n* = 1,397) (%)**	**(*n* = 1,519) (%)**	
**Three dimensions**				
BKA	52.8	43.7	37.0	42.2^*^
HLB	26.3	18.4	13.9	17.7^*^
HRS	38.2	28.3	24.0	28.0^*^
**Six domains**				
SVH	64.1	62.1	55.9	59.7^*^
PTID	25.4	22.3	23.9	23.5
PTCD	30.4	25.1	19.3	23.4^*^
SFA	72.8	63.1	58.5	62.7^*^
BMC	31.8	22.8	19.7	22.9^*^
HI	40.3	33.4	28.6	32.4^*^
Health literacy level	33.0	23.1	17.6	22.3^*^

* *P < 0.05*.

*BKA, basic knowledge and attitudes; HLB, healthy lifestyles and behaviors; HRS, health-related skills; SVH, scientific views of health; PTID, prevention and treatment of infectious diseases; PTCD, prevention and treatment of chronic diseases; SFA, safety and first aid; BMC, basic medical care; HI, health information*.

**Figure 1:**
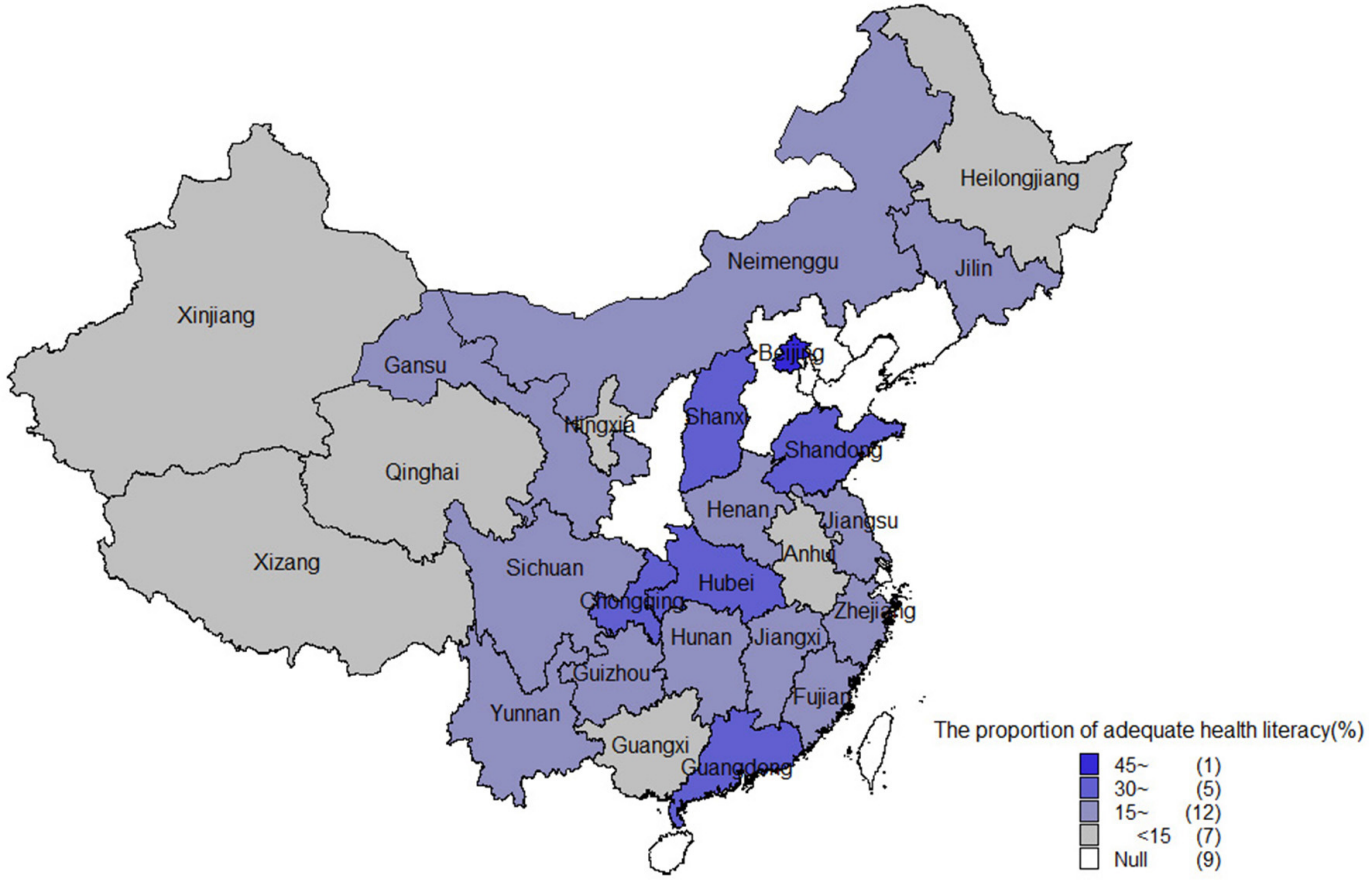
The proportion of respondents with adequate health literacy in different provinces or municipalities of China.

Figure 1 shows the provincial geographical map for the proportion of respondents with adequate health literacy. Notable geographic variation was observed in the health literacy level. The proportion of adequate health literacy ranged from 10.5% (Xinjiang) to 47.0% (Beijing).

### The Factors Influencing Health Literacy Levels in Different Regions

A further multivariate logistic regression was conducted to determine the factors of adequate health literacy. The logistic regression modeling, as shown in Table 3, demonstrated that five factors (that is, respondent's gender, education level, economic status, health status and community health education) remained significant after controlling for all the other factors. Among the five factors of health literacy, having a high education level and having a good self-rated health status were over twice as likely to have an adequate health literacy as their counterparts, with an odds ratio ranging from 2.793 to 9.458.

**Table 3:** Multiple logistic regression analysis of factors influencing adequate health literacy.

**Regions**	**Variables**	**OR**	**95% CI of OR**	
**All regions (*****n*** **= 3,482)**				
	**Gender**			
	Male	ref		
	Female	1.353	1.146	1.597
	**Education level**			
	Elementary school and below	ref		
	Junior high school	2.794	1.469	5.314
	Senior high school	6.092	3.333	11.134
	College and above	9.458	5.251	17.036
	**Economic status**			
	Poor	ref		
	Medium	1.537	1.248	1.891
	Good	1.850	1.498	2.284
	**Health status**			
	Poor	ref		
	Fair	2.793	1.534	5.083
	Good	3.003	1.672	5.395
	**Community health education**			
	0	ref		
	1–9	1.140	0.958	1.356
	≥10	1.588	1.066	2.365
**Eastern region (*****n*** **= 566)**				
	**Education level**			
	Elementary school and below	ref		
	Junior high school	1.368	0.387	4.830
	Senior high school	4.596	1.499	14.089
	College and above	5.470	1.814	16.492
	**Economic status**			
	Poor	ref		
	Medium	1.591	1.033	2.713
	Good	2.635	1.572	4.416
	**Health status**			
	Poor	ref		
	Fair	1.323	0.499	3.513
	Good	2.490	1.173	6.372
**Central region (*****n*** **= 1,397)**				
	**Gender**			
	Male	ref		
	Female	1.519	1.172	1.970
	**Education level**			
	Elementary school and below	ref		
	Junior high school	3.690	1.377	9.884
	Senior high school	5.351	2.079	13.776
	College and above	9.158	3.649	22.988
	**Economic status**			
	Poor	ref		
	Medium	1.507	1.082	2.100
	Good	1.719	1.225	2.412
	**Community health education**			
	0	ref		
	1–9	1.254	1.054	1.647
	≥10	4.331	1.910	9.817
**Western region (*****n*** **= 1,519)**				
	**Gender**			
	Male	ref		
	Female	1.331	1.012	1.750
	**Education level**			
	Elementary school and below	ref		
	Junior high school	3.630	1.044	12.625
	Senior high school	9.986	3.081	32.369
	College and above	17.551	5.516	55.843
	**Community health education**			
	0	ref		
	1–9	1.501	1.222	4.031
	≥10	3.736	1.327	10.513

This study showed that the factors affecting health literacy varied somewhat by region. High education levels, good economic status and good self-rated health status were correlated with higher health literacy levels in the eastern region. Among participants in the central region, health literacy was significantly associated with gender, education level, economic status and community health education. Female gender, high education level, and frequent community health education in the past 3 years were correlated with the higher health literacy levels of people in the western region (Table 3).

## Discussion

### Health Literacy and Its Distribution Characteristics

There are some differences in the definition of health literacy across different countries. The measurement tools and research perspectives are different, and the standards are not uniform. Therefore, it is difficult to directly compare health literacy levels among individuals in different countries. The National Assessment of Adult Literacy has reported that 36% of the United States adult population has basic or less-than-basic health literacy. Limited health literacy was especially common in Hispanic (66%), black (58%), and American Indian and Alaskan Native (48%) populations (4, 20). Nearly 19% of African American adults had a serious lack of health literacy (21).

In this study, the health literacy level was 22.3%. These findings indicated that the health literacy level of the study subjects have improved significantly in the past decade. However, less than a quarter of the participants had adequate health literacy. Furthermore, their health literacy level is still low. Previous studies have shown that the proportions of people with adequate health literacy in the United States, the United Kingdom and Japan were 64, 88.6, and 72.3%, respectively (20, 22, 23). In terms of scores on different dimensions, the participants' scores in the dimension of BKA were higher than those in the dimension of HLB. This finding demonstrated that study subjects exhibited inconsistency between knowledge and practice in health literacy, and health knowledge was not effectively translated into HLB. Under health education knowledge and belief theory, behavior change is divided into three consecutive processes: acquiring knowledge, generating beliefs, and forming behaviors. The acquisition of health knowledge is relatively easy. The transformation from knowledge into belief and then into healthy behavior is a relatively long process that is influenced by many factors, both internal and external (2).

Among the six types of health literacy, BMC literacy and chronic disease prevention literacy were relatively low, especially in the western region, which indicates the need to strengthen the understanding of scientific medical treatment, rational drug use and chronic disease prevention. In recent years, the incidence of chronic diseases in China has increased significantly, but public knowledge regarding common chronic diseases such as diabetes and high blood pressure is generally low. The phenomenon of “three high and three low” is common in the domain of PTCD and is characterized by a high incidence and prevalence of chronic diseases, a high rate of disability, low knowledge, a low control rate, and a low treatment rate. It is therefore necessary to further strengthen health education on chronic disease prevention and treatment (24).

This study showed that there were significant differences in the levels of health literacy among people in different regions, with the highest levels in the eastern region, the second-highest levels in the central region, and the lowest levels in the western region, which was consistent with the results of previous research (25). The proportion of adequate health literacy in different provinces or municipalities ranged from 10.5 to 47.0%. This might be attributable to the differences in socioeconomic status and health education resources across the sites (10, 26). These geographic disparities suggest that health practitioners and health promotion systems need to assess health literacy levels in their own settings rather than rely on national data.

A previous study showed that health literacy was a comprehensive performance of the level of social and economic development of a country or a region (15). The heterogeneity in health literacy among people in different regions was also a true reflection of the imbalance in the development of economic, cultural, and medical resources in different regions of China (13). Differences between the three regions suggest that differences in economic and cultural context may play a role in health literacy (27). This means that while national measures to improve health literacy might be appropriate for some issues, the approaches used to improve the health literacy levels of people in different regions should be adapted to local conditions.

### Factors Affecting Health Literacy and the Emphasis on Health Literacy Promotion in Different Regions

This study found that health literacy was strongly associated with education. A higher education level was independently associated with a higher health literacy level, which is consistent with the conclusions of previous studies (28–30). A better-educated person has a stronger ability to understand, analyse, and judge scientific views, making it easier to acquire and understand health literacy-related knowledge. People with lower education levels obtained less health-related information and had less experience interacting with health professionals than the general population did (31). Therefore, health education interventions should be designed based on a clear understanding of the patterns of resources available in specific groups defined by education levels.

The results of the present study revealed a significant correlation between economic status and health literacy in the eastern and central regions. This result is consistent with the findings of previous studies that showed that low socioeconomic status was correlated with low health literacy and a positive relationship between personal income and health literacy (22, 32, 33). From the perspective of economics, middle- and high-income individuals have their basic survival needs met, and so they can focus on improving their quality of life. As a result, their demand for health care services is higher than that of low-income individuals, and they can invest more attention and energy in their own health (34). Health promotion programmes may be less effective for groups with low economic status because of their poor perception of their own health status, their low use of health education resources and their limited access to relevant educational services and social support (26).

This study found a significant association between adequate health literacy and self-rated health status. This finding is consistent with those of previous studies on health literacy among office workers (35). However, we also found that self-rated health status was not significantly associated with adequate health literacy in the central and western regions. A possible explanation is that in the central and western regions, because of non-health factors such as increased economic and life pressures and less access to health education knowledge and health services, some people are seldom concerned about their own health status even if their physical condition is poor. The studies evaluating the relationship between health literacy and gender yielded mixed results. Studies by Cavanaugh and Tang Chi showed that women's health literacy level was higher than that of men, which was the exact opposite of the findings of Yan et al. (7, 25, 36). This contrast might be due to differences in the sample population and the region. This study showed that being female was predictive of increased health literacy levels. Women are more willing than men to obtain health information through various channels and are more active in obtaining health information (37). After stratification by area was performed, being female was correlated with adequate health literacy in the central and western regions, which might be due to the relative lack of health care resources in the central and western regions, and there are fewer ways for people to obtain health-related information. In the eastern region, various forms of health education information were available, and gender difference was not significant factor of the health literacy level.

Since 2011, Chinese health departments have vigorously promoted “The National Healthy Lifestyle Action,” which is based on knowledge presentation, health consultation and physical examination screening. This program is a roving health popularization activity that is conducted by urban and rural communities (38). The present study revealed that health literacy was significantly associated with community health education after adjustments were made for other factors. In the central and western regions, people who received more community health education within 3 years had higher health literacy. Popularizing health knowledge through face-to-face community health education activities is an effective way of improving the health literacy levels of people in the central and western regions. Moreover, there may be some shortcomings in health education and health promotion in those regions, and access to health knowledge is not as extensive there as it is in the eastern region. Thus, strengthening the publicity of health knowledge through various channels will be especially helpful in improving the health literacy levels of people in the central and western regions. Community health education should combine multiple approaches based on a clear understanding of the patterns of resources available among different socio-demographic groups, such as those specifically focused on disadvantaged groups, and develop the capacity of the community as a whole to act using the social resources available (26).

This study has several limitations that can be improved in further research. First, we didn't include the other six provinces in mainland China, which may have different levels of health literary from the selected 25 provinces and municipalities, considering the large diversity in different province of China. As a result, our conclusion may not be representative to the whole national level of health literacy in China. Future study may consider including all 31 provinces and municipalities to gain a full picture. Second, we did not assess the risky health behaviors (tobacco, alcohol and drug use) of the participants in this particular study, but these behaviors will be evaluated in future studies. Third, some items in this study were self-reported. We obtained data through self-reported items, such as self-rated health status. Self-reporting is prone to bias, which makes respondents more likely to provide socially desirable answers. The effect of self-reporting bias cannot be excluded in the present investigation. In addition, a cross-sectional research design was adopted in this study, which means that cause-effect conclusions could not be drawn. Despite these limitations, this study covered 25 provinces or municipalities in different regions of China and examined the level of health literacy, as well as the factors related to it. A focus was on the differences by region. This study provides a reference for developing strategies and measures to improve health literacy.

## Conclusions

The health literacy level of the participants from the 25 provinces or municipalities is relatively low compared to the developed countries, with evident heterogeneities among different regions, between urban and rural areas, and among different social groups. Tailored health education and promotion strategies are needed for different subgroups of residents.

## Data Availability Statement

The original contributions presented in the study are included in the article/supplementary material, further inquiries can be directed to the corresponding author/s.

## Ethics Statement

The studies involving human participants were reviewed and approved by Medical Ethics Committee of Central South University. Written informed consent to participate in this study was provided by the participants or their legal guardian/next of kin.

## Author Contributions

ZL performed the statistical analysis and drafted the manuscript. ZL, YT, and ZG participated in the design of the study and revision of the paper. ZL and LQ participated in data collection. All authors contributed to the article and approved the submitted version.

## Conflict of Interest

The authors declare that the research was conducted in the absence of any commercial or financial relationships that could be construed as a potential conflict of interest.

## Abbreviations

Adequate HL: Adequate health literacy
Limited HL: Limited health literacy.
